# The Mediterranean Mussel (*Mytilus galloprovincialis*) as Intermediate Host for the Anisakid *Sulcascaris sulcata* (Nematoda), a Pathogen Parasite of the Mediterranean Loggerhead Turtle (*Caretta caretta*)

**DOI:** 10.3390/pathogens9020118

**Published:** 2020-02-13

**Authors:** Mario Santoro, Erica Marchiori, Marialetizia Palomba, Barbara Degli Uberti, Federica Marcer, Simonetta Mattiucci

**Affiliations:** 1 Department of Integrative Marine Ecology, Stazione Zoologica Anthon Dohrn, Villa Comunale, 80121 Naples, Italy; 2 Department of Animal Medicine, Production and Health, University of Padua, Viale dell’Università 16, Legnaro, 35020 Padova, Italy; erica.marchiori@unipd.it (E.M.); federica.marcer@unipd.it (F.M.); 3 Department of Public Health and Infectious Diseases, Sapienza University of Rome, Piazzale Aldo Moro 5, 00185 Rome, Italy; marialetizia.palomba@uniroma1.it (M.P.); simonetta.mattiucci@uniroma1.it (S.M.); 4 Department of Animal Health, Istituto Zooprofilattico Sperimentale del Mezzogiorno, via Salute 2, Portici, 80055 Naples, Italy; barbara.degliuberti@cert.izsmportici.it

**Keywords:** anisakid nematode, mussel parasite, *Mytilus galloprovincialis*, *Sulcascaris sulcata*, Tyrrhenian Sea

## Abstract

*Sulcascaris sulcata* (Anisakidae), a pathogenic nematode of sea turtles, may cause ulcerous gastritis with different degrees of severity. Previous studies demonstrated a high prevalence of infection in the Mediterranean loggerhead turtle (*Caretta caretta*), although no data on the potential intermediate hosts of this nematode has been published thus far from the Mediterranean basin. Here, using molecular analyses, we demonstrated that the cross sections of nematode larvae observed histologically in Mediterranean mussels (*Mytilus galloprovincialis*) collected from a farm along the Tyrrhenian coast of southern Italy belong to *S. sulcata*. The BLAST analysis of sequences at the ITS2 region of rDNA and mtDNA *cox2* gene loci here obtained from samples of two Mediterranean mussels containing nematode larvae showed 100% homology with those at the same gene loci from the adults of *S. sulcata* collected from the Mediterranean Sea and deposited in GenBank. To our knowledge, this study is the first to present data on a potential intermediate host of *S. sulcata* in the Mediterranean basin and to report a nematode parasite from the Mediterranean mussel.

## 1. Introduction

The anisakid nematode *Sulcascaris sulcata* is a pathogenic parasite of the esophagus and stomach of sea turtles being able to cause ulcerous gastritis with different degrees of severity predominantly depending on the intensity of infection. *S. sulcata* infects the loggerhead turtle (*Caretta caretta*), green turtle (*Chelonia mydas*), and Kemp’s ridley turtle (*Lepidochelys kempii*) in the Mediterranean and Caribbean Seas, and the South Atlantic, Western Atlantic, and Western Pacific Oceans [1]. 

Berry and Cannon [2] demonstrated experimentally that hatchling loggerhead turtles become infected by ingesting scallops infected with fourth stage larvae *S. sulcata*. Larvae attach at the base of the esophagus where four molts occur about three weeks after infection and mature to adults in at least 5 months. Adult parasites live in the stomach of sea turtles and eggs are shed in the marine environment with host feces. Two molts occur in the egg until the development of the third stage larva, which spontaneously hatches and develops in bivalve and gastropod mollusks to fourth stage larva after 3–4 months. The duration of the natural cycle could be of two years [2].

Recently, we described the pathological changes caused by *S. sulcata* in two loggerhead populations inhabiting the Tyrrhenian and the Adriatic Seas in the Mediterranean basin [1]; however, no data exists on its intermediate hosts in this geographical area. Published data of intermediate hosts of *S. sulcata* exists only for Australian and American waters where at least 17 taxa of mollusks have been found infected or have been experimentally infected including six gastropods and 11 bivalves (listed in Table 1).

While studying the occurrence of protozoan parasites in the Mediterranean mussel (*Mytilus galloprovincialis*) along the coast of Campania region of southern Italy, larval forms of nematodes were observed histologically in the tissues of Mediterranean mussels. Herein, using molecular analysis, we report for the first time the occurrence of larvae of *S. sulcata* in Mediterranean mussels from the Tyrrhenian Sea. 

## 2. Results

A total of five (1.4%) individual Mediterranean mussels collected on February (one mussel), May (one mussel), July (two mussels), and August (one mussel) were histologically positive to one (n = 3), two (n = 1), and three (n = 1) cross sections of nematode larvae, respectively. Cross sections of larvae (n = 4) measured in mean 290.5 μm (range: 178 to 430.9) x 202.5 μm (range: 149.4 to 292.8). Larvae were encysted within the foot of Mediterranean mussels extending to the digestive gland and revealed host inflammatory reaction in all cases. In most cases, larvae appeared to be viable and were surrounded by well-defined hemocytic capsules.

**Figure 1:**
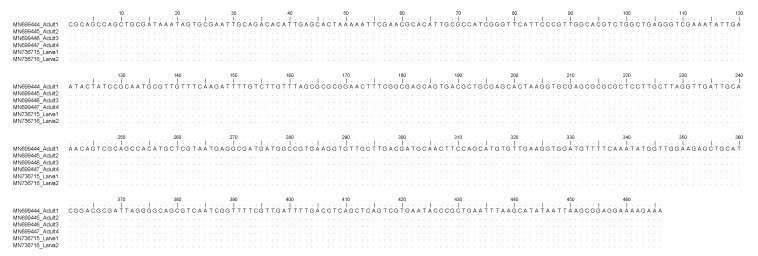
Nucleotide sequence alignment of the nDNA ITS2 obtained from *Sulcascaris sulcata* larvae (Accession No. MN736715, MN736716) in comparison with those previously identified as *S. sulcata* adults (Accession No. MN699444, MN699445, MN699446, MN699447). Dots indicate identity with the consensus sequence.

**Figure 2:**
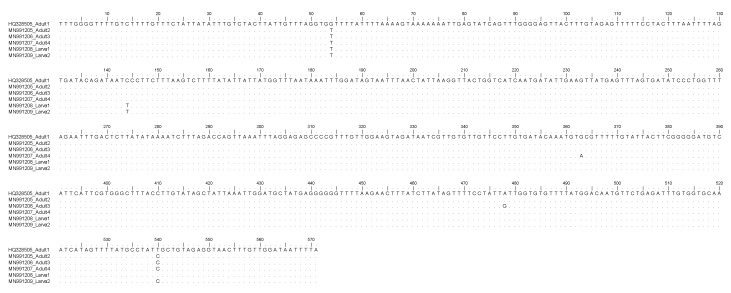
Nucleotide sequence alignment of the mtDNA *cox2* obtained from *Sulcascaris sulcata* larvae (Accession No. MN991208, MN991209) in comparison with those previously identified as *S. sulcata* adults (Accession No. HQ328505, MN991205; MN991206; MN991207). Dots indicate identity with the consensus sequence.

The BLAST analysis of the ITS2 of rDNA and mtDNA *cox2* sequences produced here from samples of two Mediterranean mussels containing nematode larvae showed 100% homology with those of adult stages of *S. sulcata* from the Mediterranean Sea, previously deposited in GenBank (Figure 1 and Figure 2). Sequences obtained in the present study were deposited in GenBank under accession numbers MN736715.1 and MN736716.1 for ITS2 and MN991208 and MN991209 for *cox*2. 

## 3. Discussion

The Mediterranean mussel has been intensively studied for pathogens in the whole Mediterranean as well as the Campanian coastal areas [19,20,21], but to date, our study describes the first finding of a parasite nematode in this bivalve species. According to McElwain et al. [22], parasitic nematodes are uncommon in marine bivalves. In mussels, the only data is by Lauckner [23], reporting in North Atlantic an infection by an anisakid larva thought to be *Phocanema* (*Pseudoterranova*) *decipiens* in a North Atlantic *Mytilus edulis*. 

In the Mediterranean Sea, the occurrence of *S. sulcata* in loggerhead turtles seems to be limited to its eastern basin and the Tyrrhenian Sea [1,24]. Recently, we observed that all *Sulcascaris* positive loggerhead turtles from the Tyrrhenian came from the coastal sites located between Castel Volturno and the Naples Gulf (it includes also Monte di Procida), where all Mediterranean mussel farms and wild bivalve beds, registered along the Campania coast are concentrated [1]. According to Berry and Cannon [2], it is plausible to think that the farmed Mediterranean mussels may have been infected by filtering seawater contaminated with *S. sulcata* eggs and/or larvae laid with feces by an infected loggerhead turtle while feeding on farm ropes of Mediterranean mussels. 

Adult individuals of loggerhead turtles are frequently observed feeding on Mediterranean mussel farm ropes along the coast of Campania. Ingestion of large amounts of Mediterranean mussels by loggerhead turtles was confirmed by our unpublished observations at the post-mortem examination of large individuals stranded along the coast of Campania. In those loggerhead turtles, the remains of Mediterranean mussels in the gastrointestinal tract commonly occurred in the presence of *S. sulcata* individuals within the gastric lumen. This particular feeding habit, rarely observed in other Mediterranean basins [25,26,27], could favor the increase of the incidence of infection with *S. sulcata* in Mediterranean mussels along the coast of Campania.

Regarding the site of infection, all larval forms of *S. sulcata* occurred within the foot of the Mediterranean mussels showing an encapsulation-type inflammatory response [28,29]. In Australian and American waters, fourth-stage larvae occurred most commonly within the adductor muscle and gonads of its molluscan hosts (Table 1). The only report of *S. sulcata* larvae within the foot was in the bivalve *Spisula solidissima* from the Atlantic coast of United States where larvae were associated with necrosis and hemocytic infiltration [16]. It has been documented that under the influence of environmental changes and the associated degradation of preferred habitats, host switching by parasites may increase [30], and parasite larvae may adapt their tropism changing the intermediate host species [31]. In particular, in the absence of preferred intermediate hosts and rich presence of definitive hosts, other marine mollusks might play a prominent role in the parasite transmission [32], and parasites may adapt in different way to the new hosts. Most of the marine bivalves found as intermediate hosts in Australian and American waters are Pectinidae of commercial importance (Table 1). Contrarily to that observed in other Mediterranean areas (for example the Adriatic Sea), Pectinidae mollusks along the Tyrrhenian coast are rare and do not represent usual food items for loggerhead turtles [33]. The significantly higher values of *S. sulcata* infection among loggerhead turtles feeding in Adriatic compared to Tyrrhenian have been attributed to the differences of regional habitats supporting higher abundance and diversity of mollusk intermediate hosts [1], and it is plausible to suppose that other intermediate hosts for *S. sulcata* may occur in other Mediterranean areas where this nematode is known to be widespread.

It is unknown if the adductor muscles of examined Mediterranean mussels were also infected because routinely it has been left on the internal surface of the valves, and it has not been processed by histological examination [34]. Further studies to evaluate the occurrence of nematode larval forms within the adductor muscle are warranted to understand if the prevalence of infection for *S. sulcata* larvae in Mediterranean mussels from this basin was underestimated and if it was an occasional finding.

Here, the molecular study based on the sequence analysis at the ITS2 region of rDNA and the mtDNA *cox2* gene loci, allowed the first molecular identification of the larval nematodes as belonging to *S. sulcata* in Mediterranean mussels. In particular, sequences analysis of the mtDNA *cox2* generally allows the identification of larval stages of anisakid nematodes included in the genera *Anisakis, Pseudoterranova,* and *Contracaecum* [35]. Additionally, the same primers used for the anisakid parasites permitted, in recent years, the direct sequencing of several adult nematodes belonging to the species *S. sulcata* obtained from loggerhead turtles stranded along the Mediterranean coast (Mattiucci, unpublished data). As a consequence, the direct alignment of the mtDNA *cox2*, here obtained on the two samples with respect to the sequences of adult specimens, have permitted the identification of those ascaridoid larval stages as corresponding to *S. sulcata*. On the other hand, mtDNA *cox2* allowed also the identification of *Anisakis pegreffii* larvae in human granuloma tissues, surgically removed and formalin-embedded in human cases of anisakiasis [35]. Analogously, the direct sequences analysis of the ITS2 region of rDNA was also previously performed on adults of *S. sulcata* (Marcer, unpublished data) allowing the identification of its larvae in the same DNA samples. However, in the case of ITS2 region of rDNA, being generally a conservative gene locus in anisakid nematodes [35], no genetic variation was observed between adult and larval specimens of *S. sulcata*, here examined (Figure 1), whereas the mtDNA *cox2* presented, at the intraspecific level, variation at non-diagnostic nucleotide positions (Figure 2); this finding is in accordance with the high polymorphism generally detected at this gene locus at the intraspecific level in several heteroxenous anisakid nematodes, such as those observed in species of the genus *Anisakis* [35].

This result represents the first report of *S. sulcata* invading tissues of Mediterranean mussels, and it strongly suggests the Mediterranean mussel as an intermediate host for *S. sulcata* in the Tyrrhenian Sea. The Mediterranean mussel is the most economically important shellfish produced and consumed in the Western Mediterranean. The Gulf of Naples in southern Italy is among the most important production sites of the Mediterranean mussel in Italy with about 4170 tons per year [36]. Infection of Mediterranean mussels by larval nematodes may cause economic losses due to the prohibition of its marketing. Experimental studies performed on fish, chickens, and cats suggest that infection by *S. sulcata* may occur only in sea turtles as definitive hosts [2]; however since *S. sulcata* is a strictly related species to zoonotic anisakid parasites [35,37], the concern regarding public health should not be underestimated.

## 4. Materials and Methods

### 4.1. General Data

From February to August 2018, a total of 363 individual Mediterranean mussels (>5 cm in length) were collected from a mussel farm located in Monte di Procida (Naples municipality), under the framework of a control program for protozoan surveillance in the Campania region of southern Italy. The farm was located in the Tyrrhenian Sea about 800 m offshore the Campanian coastline on the sand bottom at a depth of 30 m; the Mediterranean mussels were cultured on ropes suspended from floating buoys at a depth of 5–12 m.

### 4.2. Histological Procedures

Immediately after collection, the mussels were stored at 4 °C, and within 12 h they were processed by histological examination for pathogen detection [34]. Transverse sections of the mantle, gonad, digestive gland, gills, and foot were obtained in a single slice through the midbody, fixed in buffered 10% formalin, and sectioned at a thickness of 5 mm. Sections were stained with hematoxylin and eosin for histologic evaluation.

### 4.3. Anisakid DNA Extraction and Molecular Identification

To confirm the identity of larval nematodes observed by histological analysis, we performed the extraction of nematodes’ DNA from microtome slices of five paraffin-embedded infected Mediterranean mussels. The removal of paraffin and extraction of DNA was carried out using NucleoSpin^®^ Tissue Macherey–Nagel kit (GmbH&Co., Germany) according to the manufacturer’s instructions for paraffin embedded tissues.

Amplification of the ITS2 region of rDNA was carried out on the samples using the primers D (5′-GAGTCGATGAAGAACGCAG-3′) and reverse B1 (5′-GAATCCTGGTTAGTTTCTTTTCCT-3′) [38] in a 50 µL reaction, comprising 5 µL DNA, 1.5 mM MgCl_2_, 0.2 mM dNTPs (MBI Fermentas, Germany), 1X PCR buffer, 0.3 μM each of forward and reverse primer, and 1 U Platinum Taq DNA Polymerase (Invitrogen). Molecular biology grade water was added up to the final volume. The mixture was amplified with an initial activation step at 94 °C for 2 min, followed by 35 cycles of denaturation at 94 °C for 30 s, annealing at 58 °C for 30 s, DNA extension at 72 °C for 35 s, and a final extension step of 72 °C for 5 min.

For sequencing the mitochondrial cytochrome C oxidase subunit II (*cox2*) gene, PCR amplification was performed using the primers 211F (5′-TTTTCTAGTTATATAGATTGRTTTYAT-3′) and 210R (5′-CACCAACTCTTAAAATTATC-3′) [39,40]. PCRs were carried out in a 50 µL volume containing 30 pmol of each primer, MgCl_2_ 2.5 mM (Amersham Pharmacia Biotech. Inc., Piscataway, NJ), 1× PCR buffer (Amersham Pharmacia Biotech. Inc., Piscataway, NJ, USA), DMSO 0.08 mM, dNTPs 0.4 mM (Sigma-Aldrich, St. Louis, MO, USA), 5 U of Taq Polymerase (Amersham Pharmacia Biotech. Inc., Piscataway, NJ, USA), and 10 ng of total DNA. PCR temperature conditions were the following: 94 °C for 3 min (initial denaturation), followed by 34 cycles at 94 °C for 3 min (denaturation), 46 °C for 60 s (annealing), 72 °C for 90 s (extension), followed by post-amplification at 72 °C for 10 min.

The PCR products were resolved in 2% agarose gel (expected fragments length: 500 bp of ITS2 and 629 bp of mtDNA *cox2* gene) and sequenced in both directions by Macrogen (Macrogen Europe, The Netherlands). The chromatograms were corrected using the software ChromasPro version 2.4.3 (Technelysium Pty Ltd, Australia). The consensus sequences were assembled with the program SeqMan available in the DNAstar package. The consensus sequences were compared with the non-redundant database available in the GenBank using the software BLASTn [41].

## Figures and Tables

**Table 1:** Marine molluscan hosts for larval forms of *Sulcascaris sulcata* updated by Lichtenfels et al. [3].

Molluscan Host	Natural/Experimental Infection	Geographical Sites	References
Bivalves			
*Amussium balloti*	Natural	Queensland, Shark Bay (Western Australia)	[2,4,5]
*Argopecten gibbus*	Natural	Florida, Southeast coast of United States	[6,7,8,9,10]
*Argopecten irradians*	Natural	North and South Carolina	[7,8,11,12]
*Chlamys* sp.	Natural	Queensland	[4]
*Melina ephippium*	Experimental	Australia	[2]
*Pecten* sp.	Natural	North Carolina	[13]
*Pecten ziczac*	Natural	Southeastern Brazil	[14]
*Pinna menkei*	Natural	Moreton Bay, Australia	[15]
*Pinctada* sp.	Experimental	Australia	[2]
*Spisula solidissima*	Natural	Massachusetts to North Carolina	[7,8,16,17,18]
*Spondylus ducalis*	Natural	Bundaberg, Australia	[15]
Gastropods			
*Busycon canaliculatum*	Natural	Virginia	[8,17]
*Cypraea tigris*	Natural	Great Barrier Reef, Australia	[15]
*Fasciolaria lilium hunteria*	Natural	Florida	[3]
*Lunatia heros*	Natural	Virginia	[8,17]
*Pleuroploca gigantea*	Natural	Florida	[3]
*Polinices sordidus*	Experimental	Australia	[2]
